# Efficacy and safety of tenofovir disoproxil fumarate versus entecavir in the treatment of acute-on-chronic liver failure with hepatitis B: a systematic review and meta-analysis

**DOI:** 10.1186/s12876-023-03024-7

**Published:** 2023-11-13

**Authors:** Neng Wang, Sike He, Yu Zheng, Lichun Wang

**Affiliations:** 1 Center of Infectious Disease, West China Hospital, Sichuan University, Chengdu, 610041 Sichuan People’s Republic of China; 2 West China School of Medicine, Sichuan University, Chengdu, Sichuan People’s Republic of China

**Keywords:** Hepatitis B virus, Acute-on-chronic liver failure, Therapy, Meta-analysis, Tenofovir, Entecavir

## Abstract

**Background:**

Oral nucleoside (acid) analogues (NAs) are recommended for patients with acute-on-chronic liver failure (ACLF) associated with hepatitis B virus (HBV-ACLF). The efficacy and safety of tenofovir (TDF) and entecavir (ETV) in these patients remain unclear.

**Methods:**

A comprehensive literature search in PubMed, Web of Science, The Cochrane Library, and Embase database was conducted to select studies published before December 2022 on TDF or ETV for HBV-ACLF. The primary outcomes were survival rates at 4, 12, and 48 weeks. Secondary outcomes were virologic and biochemical responses, serum antigen conversion, liver function score, and safety.

**Results:**

Four prospective and one retrospective cohort studies were selected. The overall analysis showed comparable survival rates at 4, 12, and 48 weeks for all patients receiving TDF or ETV (4-week: RR = 1.17, 95% CI: 0.90–1.51, *p* = 0.24; 12-week: RR = 1.00, 95% CI: 0.88–1.13, *p* = 0.94; 48-week: RR = 0.96, 95% CI: 0.58–1.57, *p* = 0.86). Child-Turcotte-Pugh (CTP) score and model for end-stage liver disease (MELD) score at 12 weeks were comparable in both groups but lower than baseline (CTP: SMD = -0.75, 95% CI:-2.81–1.30, *p* = 0.47; MELD: SMD = -1.10, 95% CI:-2.29–0.08, *p* = 0.07). At 48 weeks, estimated glomerular filtration rate (eGFR) levels were found to decrease to different degrees from baseline in both the TDF and ETV groups, and the decrease was greater in the TDF group than in the ETV group. No significant differences were found in biochemical, virologic response, and serum antigen conversion between the two groups during the observation period.

**Conclusion:**

TDF treatment of HBV-ACLF is similar to ETV in improving survival, liver function, and virologic response but the effects on renal function in two groups in the long term remain unclear. More and larger long-term clinical trials are required to confirm these findings.

**Supplementary Information:**

The online version contains supplementary material available at 10.1186/s12876-023-03024-7.

## Introduction

Acute-on-chronic liver failure (ACLF) occurs in patients with chronic liver disease and is characterized by acute liver injury, such as jaundice and coagulopathy [1]. The global prevalence of ACLF is higher than 30%, and the highest prevalence in South Asia is approximately 65% in patients with decompensated cirrhosis [2]. The deaths of these patients within 3 months were mainly due to multisystem organ failure and severe infection [3, 4]. In Asia, ACLF is mainly caused by hepatitis B virus (HBV) infection and has a mortality rate of 50–90% [1, 5]. Liver transplantation is considered the ultimate treatment for ACLF. Unfortunately, liver transplantation is limited by a lack of donor organs, high cost, use of immunosuppressants, and the potential risk of serious complications [6].

HBV-ACLF exhibits different clinical features from other etiologically related ACLF. Recurrence of hepatitis B, superimposed infection with other hepatitis viruses (A or E), and mutations in resistance to antiviral therapy are common triggers of high mortality [7–9]. For HBV-ACLF, the guidelines recommend early use of effective antiviral nucleoside/nucleotide analogues (NAs), such as tenofovir disoproxil fumarate (TDF) and entecavir (ETV) [10–12]. NAs can effectively inhibit viral reverse transcriptase and reduce the HBV load in the blood, thereby reducing secondary inflammation and promoting hepatocyte regeneration and disease recovery [6, 11]. NAs have fewer side effects, a low incidence of adverse reactions, and are safe to use. However, complications such as renal insufficiency and bone calcium and phosphorus metabolism disorders may occur due to the long-term use of NAs, especially in older adults with comorbidities [10, 12].

Many studies have reported the efficacy of ETV on the survival of patients with HBV-ACLF. Studies have shown no difference in short-term survival after 12 weeks of ETV treatment compared to controls without antivirals [13, 14], while other studies have reported improved survival [15]. Unlike ETV, data on the efficacy of TDF for HBV-ACLF are limited. Comparison of the efficacy of HBV-ACLF and the clinical choice of these two drugs remains controversial. To the best of our knowledge, there are no systematic reviews in the literature aimed at investigating the efficacy and safety of TDF versus ETV in the treatment of patients with HBV-ACLF.

## Materials and methods

### Literature search

We performed this systematic review and meta-analysis according to the Preferred reporting items for systematic reviews and meta-analyses (The PRISMA statement) [16]. Two independent researchers (NW and SKH) searched PubMed, Web of Science, Cochrane Library, ClinicalTrial.gov, and Embase. Articles were restricted to publication until December 2022. The following combinations of keywords and Boolean operators were used in the MeSH and free-text searches: hepatitis B virus infection or HBV infection; acute-on-chronic liver failure or ACLF or HBV-ACLF; nucleoside or nucleotide analogues or Nuc or NA; tenofovir or TDF; and entecavir or ETV. The detailed search strategy is shown in Supplementary Table 1 (Additional file 1). Two researchers searched independently by title and abstract. The search results were then combined to perform an initial screening of desired articles. The full text was then read to screen for articles that met the inclusion criteria. Baseline and endpoint parameters were extracted from each group.

### Inclusion and exclusion criteria

Inclusion criteria: (1) meeting the Asia Pacific Association for the Study of the Liver (APASL) ACLF criteria (APASL-criteria) for serum bilirubin ≥5 mg/dL, international normalized ratio ≥ 1.5 or prothrombin activity < 40% in patients with previously diagnosed or undiagnosed chronic liver disease within 4 weeks with ascites or encephalopathy [17]; (2) age between 18 and 65 years; (3) oral treatment with TDF or ETV; (4) full-text extractable data related to the outcome metric. Exclusion criteria. (1) duplicate or unavailable publications; (2) single arm only, no comparison of TDF and ETV groups; (3) combination of antiviral therapy with other drugs during treatment, no drug control group; (4) other causes of chronic liver failure, such as drug-related liver injury, autoimmune liver disease, alcoholic liver disease and inherited metabolic diseases; malignancies and severe haematological abnormalities; (5) studies must have objective outcome indicators or they will be excluded from this analysis.

### Data extraction

Two independent researchers (YZ and NW) performed all data extraction and statistics. Seven parameters were extracted: survival rate, HBV-DNA level, HBV-DNA clearance rate, serum surface antigen conversion, Child-Turcotte-Pugh (CTP) score, model for end-stage liver disease (MELD) score, and safety. When the data was not provided directly in texts, GetData Graph Digitizer (version 2.26) would be used for extracting data from graphs. Differences in retrieval results or differences in opinion were resolved by discussion among all participants. If two investigators disagreed, a third author (LCW) was consulted.

### Quality assessment

Two reviewers (SKH and NW) independently assessed the qualities of eligible studies by using the Newcastle–Ottawa Scale (NOS) [18], where scores of 1 to 3, 4 to 6, and 7 to 9 were considered low, medium, and high quality, respectively.

### Outcome assessment

We focused on patient survival at 4, 12, and 48 weeks for prognostic assessment. Secondary endpoints included virologic and biochemistry response, serum surface antigen conversion, CTP score, MELD score, and safety.

### Statistical analysis

Data analysis was performed using Stata (version 14.0). The results for dichotomous variables were assessed and expressed as risk ratios (RRs) and 95% confidence intervals (CIs). In addition, standardized mean differences (SMDs) and 95% CIs were selected for continuous variables due to the large differences in means between studies. Statistical heterogeneity was assessed with χ^2^ and I^2^ tests. Values of *p* < 0.10 or I^2^ > 50% were considered statistically significant when combined with the results of the random-effects model. Begg’s test or Egger’s test was performed to assess the publication bias. Publication bias was considered statistically significant if the *p*-values were < 0.05. All statistical analyses were conducted using the Review Manager 5.4 and the Stata 14.0.

## Results

### Basic characteristics of the included studies and risk of bias evaluation

**Fig. 1:**
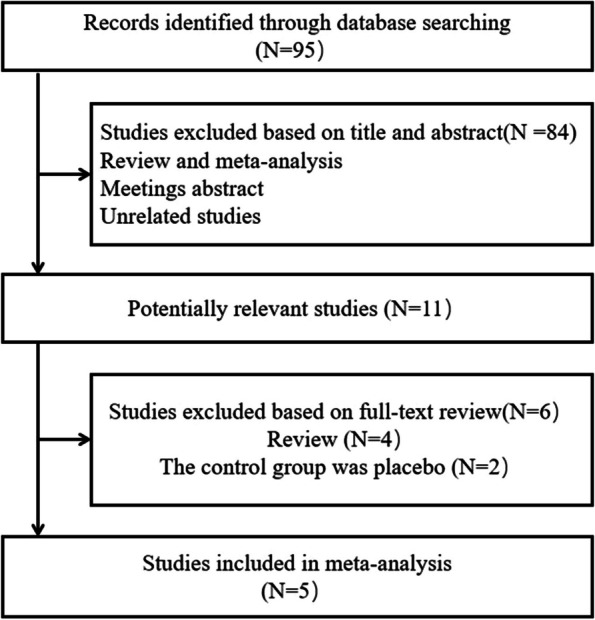
Identification process for eligible studies. The 95 studies initially identified from our electronic search met the inclusion criteria and were included in this meta-analysis

The results retrieved 95 articles, and finally, a total of five studies [19–23] with 272 patients were included. The article screening process is shown in Fig. 1. The five studies were divided into four prospective cohort studies [19, 20, 22, 23] and one retrospective cohort study [21]. The details of the five articles are shown in Table 1. Risk of bias analysis was performed for the included studies, and the risk of bias was acceptable for all studies based on quality analysis, as shown in Table 2.

**Table 1:** Characteristics of the included cohort studies

Studies	Year	Study design	Country	Intervention(TDF/ETV)	Age, Mean (SD)(TDF/ETV)	Sample size (TDF/ETV)	Follow-up time (weeks)	Outcome
Hossain et al. [17]	2021	Prospective cohort	Bangladesh	300 mg/d 0.5 mg/d	43.8 ± 13.1 44.2 ± 12.3	16/16	12	①Survival rate ② HBV-DNA clearance rate ③CTP score ④MELD score
Zhang et al. [13]	2020	Prospective cohort	China	NR	44.33 ± 15.87 45.97 ± 14.10	39/39	24	①Survival rate ② HBV-DNA levels ③Serum surface antigen conversion
Zhang et al. [16]	2021	Prospective cohort	China	300 mg/d 0.5 mg/d	38.87 ± 7.95 43.90 ± 9.91	23/42	24	①Survival rate ② HBV-DNA clearance rate ③Serum surface antigen conversion ④MELD score ⑤Safety
Li et al. [14]	2021	Prospective cohort	China	300 mg/d 0.5 mg/d	41 ± 12.64 39.72 ± 9.13	10/20	48	①Survival rate ②HBV-DNA levels ③MELD score ④Safety
Wan et al. [15]	2019	Retrospective cohort study	China	300 mg/d 0.5 mg/d	43.54 ± 11.13 50.71 ± 10.7	32/35	12	①Survival rate ②HBV-DNA levels ③HBV DNA clearance rate ④Serum surface antigen conversion ⑤CTP score ⑥MELD score ⑦Safety

*TDF* Tenofovir, *ETV *Entecavir, *NR* Not reported

**Table 2:** Quality evaluation of the included cohort studies via the NOS

Studies	Representativeness of the exposed cohort	Selection of the nonexposed cohort	Ascertainment of exposure	Outcome not present at baseline	Control for age and sex	Control for other confounding factors	Assessment of outcome	Enough long follow-up duration	Adequacy of follow-up of cohort	Total
Hossain et al. [17]	⁎	⁎	⁎	⁎	⁎		⁎			6
Zhang et al. [13]		⁎	⁎	⁎	⁎	⁎	⁎		⁎	7
Zhang et al. [16]	⁎	⁎	⁎	⁎	⁎		⁎	⁎	⁎	8
Li et al. [14]	⁎	⁎		⁎	⁎	⁎	⁎	⁎	⁎	8
Wan et al. [15]	⁎	⁎	⁎	⁎	⁎		⁎	⁎	⁎	8

*TDF* Tenofovir, *ETV* Entecavir, *NR* Not reported

### Survival rate

**Fig. 2:**
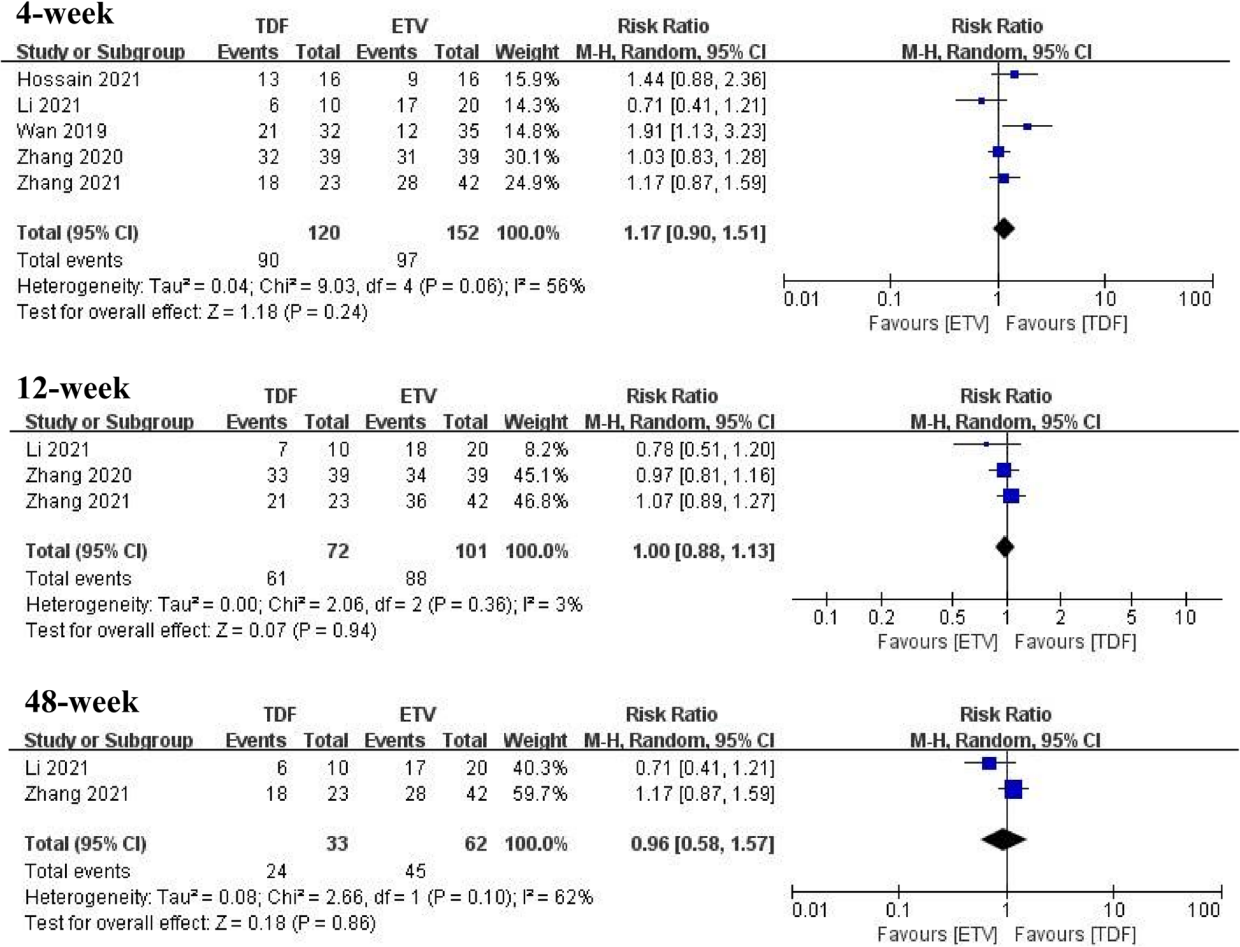
Survival rates at 4, 12, and 48 weeks for TDF and ETV in all included studies

Three of the included studies [19, 20, 22] reported the 4-week survival rates of patients and found that TDF did not significantly improve the 4-week survival rates compared with the ETV group (RR = 1.17, 95% CI: 0.90–1.51, *p* = 0.24). Five studies [19–23] provided data on 12-week survival rates, and the combined analysis found no significant difference between the two groups (RR = 1.00, 95% CI: 0.88–1.13, *p* = 0.94). Two studies [20, 22] comparing 48-week survival rates with HBV-ACLF showed that there was no significant difference between the two groups (RR = 0.96, 95% CI: 0.58–1.57, *p* = 0.86) (Fig. 2).

### Effect of antiviral therapy on HBV-DNA

**Fig. 3:**
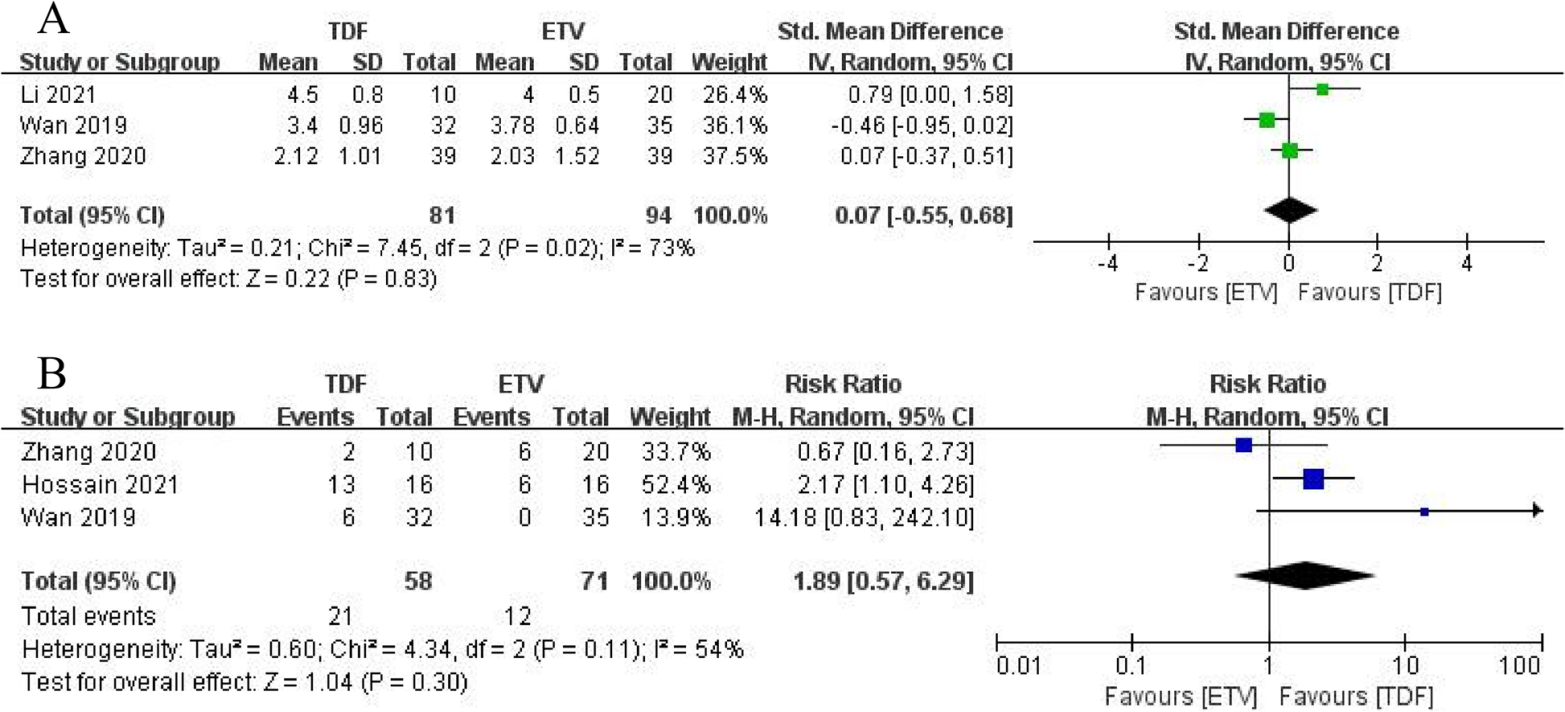
**A** Reduced HBV DNA levels of TDF and ETV at 2 weeks. **B** HBV DNA clearance rate of TDF and ETV at 12 weeks

Three studies [19–21] compared HBV-DNA levels between the two groups at 2 weeks and found no significant difference in HBV-DNA levels between TDF and ETV (SMD = 0.07, 95% CI:-0.55–0.68, *p* = 0.83) (Fig. 3A). Three studies [21–23] reported rates of unmonitored HBV-DNA at 12 weeks and demonstrated that TDF was not effective in improving the HBV-DNA clearance rate in patients compared to ETV (RR = 1.89, 95% CI:0.57–6.29, *p* = 0.30) (Fig. 3B).

### Serum surface antigen conversion

In the study by Wan et al. [21], 2 and 4 HBeAg+ patients in the ETV group and the TDF group survived for 3 months. Of these patients, none in the ETV group (0%; 0/2) and 4 in the TDF group (100%; 4/4) had HBeAg loss (*p* = 0.067). None had HBeAg serologic conversion at 3 months. Zhang et al. [22] reported no surface antigen loss in the two groups at week 48. There was 1 case of HBeAg serologic conversion in each group, and the time to conversion was 12 and 48 weeks in the TDF and ETV groups, respectively.

### Biochemical response

**Fig. 4:**
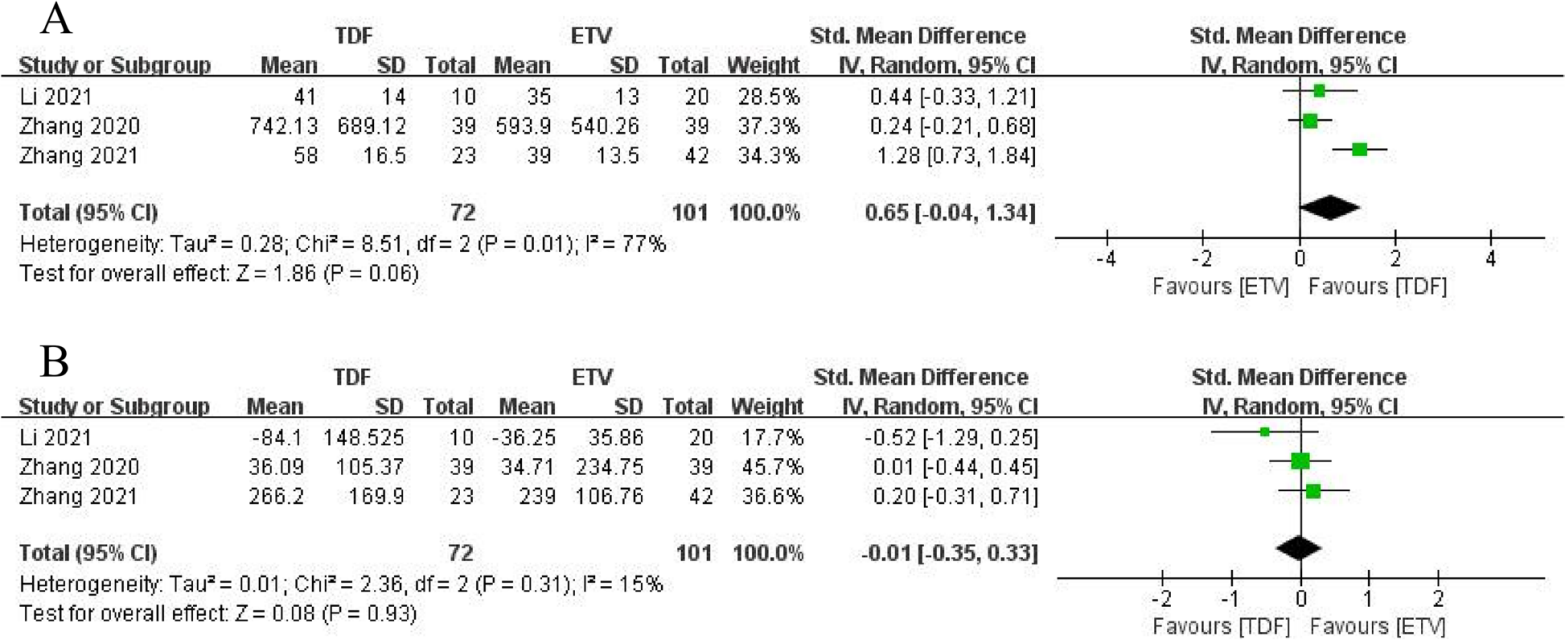
**A** Alanine aminotransferase levels at 4 weeks after TDF and ETV therapy. **B** Total bilirubin levels at 4 weeks after TDF and ETV therapy

Regarding changes in liver function, three studies [19, 20, 22] examined the changes in alanine aminotransferase (ALT) and total bilirubin (TBiL) at 4 weeks in the TDF and ETV groups. The results showed improvement in ALT and TBiL compared to baseline levels, but there was no remarkable difference between the two groups (ALT: SMD = 0.65, 95% CI: − 0.04 − 1.34, *p* = 0.06; TBiL: SMD = − 0.01, 95% CI: − 0.35 − 0.33, *p* = 0.93). Only one study [22] reported ALT and TBiL at 12 and 48 weeks and found that TDF did not improve ALT and TBiL levels in patients with ACLF compared to ETV (*p* > 0.05). More details can be found in Fig. 4.

### CTP score and MELD score

**Fig. 5:**
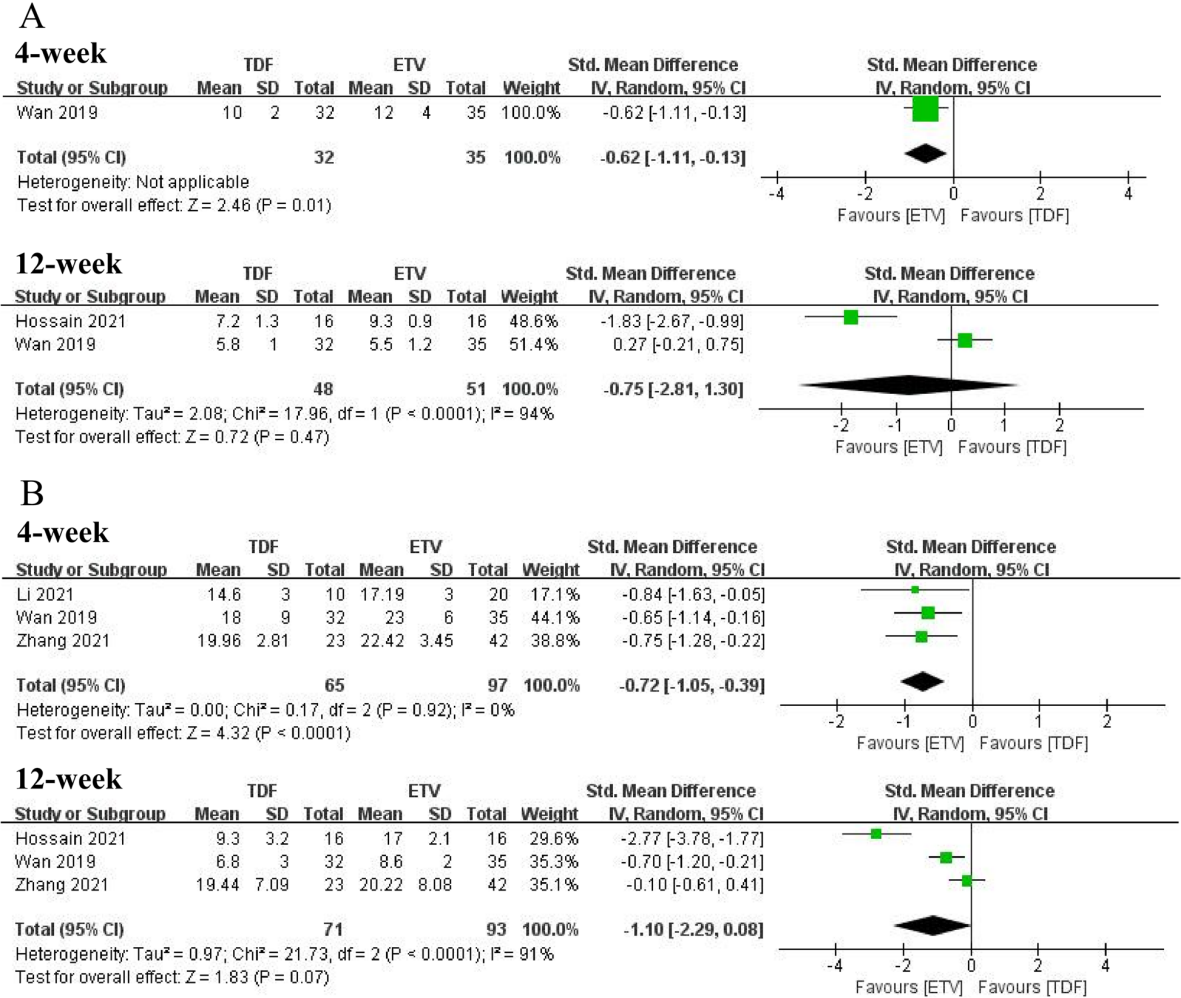
**A** CTP score for TDF and ETV therapy. **B** MELD score for TDF and ETV therapy

The CTP and MELD scores at 4 weeks were reported in one study [21] and three studies [20–22], respectively. Both CTP and MELD scores were in two separate groups (p > 0.05 between baselines, comparable), and after 4 weeks of treatment, ETV failed to improve CTP scores or MELD scores, whereas TDF improved CTP and MELD scores (CTP: SMD = − 0.62, 95% CI: − 1.11 − − 0.13, *p* = 0.01; MELD: SMD = − 0.72, 95% CI:-1.05 − − 0.39, *p* < 0.0001). The CTP and MELD score at 12 weeks were separately mentioned in two studies [21, 23] and three studies [20–22], and TDF did not improve the two scores in patients with ACLF compared with ETV (CTP: SMD = -0.75, 95% CI:-2.81–1.30, *p* = 0.47; MELD: SMD = -1.10, 95% CI: − 2.29 − 0.08, *p* = 0.07). Forest plots are presented in Fig. 5. Only one study [22] reported the MELD score at 24 weeks and there was no significant difference between the two groups (*p* > 0.05).

### Safety of TDF and ETV in HBV-ACLF

Two studies by Zhang et al. and Li et al. [20, 22] provided changes in estimated glomerular filtration rate (eGFR) over 4 weeks, but no significant differences between the two groups were found in their respective reports. Li et al. [20] focused on eGFR at 4, 12 and 48 weeks and found that the eGFR decreased differently from baseline at week 4 in the TDF and ETV groups, and the decrease was greater in the TDF group than in the ETV group (− 5.83 vs − 4.75 mL/min/1.73m^2^). However, it remained unclear whether the difference in nephrotoxicity is statistically significant. The study by Zhang et al. [19] reported increased serum creatine and cystine C in both TDF and ETV groups but there was no significant difference between them. In addition, Wan et al. [21] did not find patients with severe lactic acidosis or renal impairment attributable to ETV or TDF treatment at the 3-month follow-up, while Zhang et al. and Li et al. [20, 22] did not observe renal-related adverse events, severe renal adverse events, or proximal tubulopathy events during the 48-week follow-up, and patients tolerated antiviral therapy well.

### Sensitivity analysis and publication bias

**Fig. 6:**
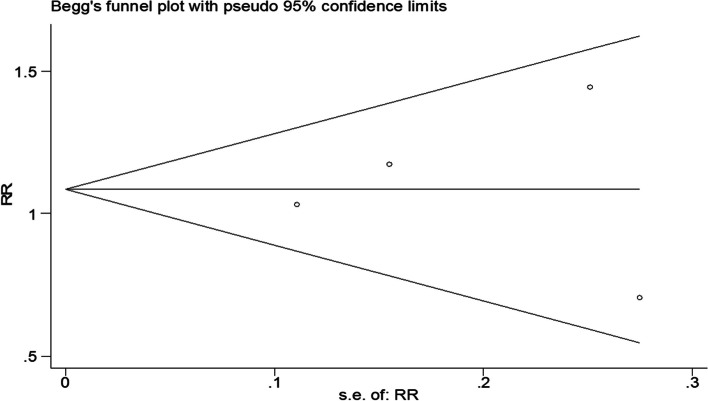
Begg’s test of survival rate at 12 weeks. The horizontal line in the funnel plot indicates the fixed effects summary estimates, while the diagonal line indicates the expected 95% confidence interval given the standard errors, assuming no heterogeneity between studies. Publication bias was not observed in studies using Egger’s (*p* = 0.91) test, suggesting no evidence of publication bias

We noted large heterogeneity in the MELD score and CTP score at 12 weeks (*I*^2^ = 94.7% and *I*^2^ = 91.3%). Sensitivity analysis showed that the study by Hossain et al. was the main source of heterogeneity in the two combined analyses. By removing this study and combining the analyses again, no substantial changes were found in the above results, indicating good stability of the meta-analysis results. In addition, the I^2^ value of the 12-week survival forest plot decreased from 55.7 to 28.3% after removing data such as the 12-week survival rate in Wan et al.. No noteworthy publication bias was found in Begg’s test and Egger’s test, which indicates that there was no significant publication bias (*p* = 0.91 in Begg’s test) (in Fig. 6).

## Discussion

ACLF was first proposed in 1995 and is now considered a life-threatening syndrome that differs from simple decompensated cirrhosis in clinical, pathophysiological, and prognostic aspects [24]. There are some differences and regional variations between the East and West regarding the underlying chronic liver disease and acute injury in ACLF. In Asia, most cases of ACLF are caused by the reactivation of hepatitis B superimposed on underlying chronic liver disease (not necessarily cirrhosis) [17, 25]. Therefore, oral NAs therapy provides a rational method for treating HBV-ACLF, especially in Asia, by suppressing viral DNA and reducing the development of hepatic necroinflammation [26]. Our study is the first meta-analysis designed to assess the efficacy and safety of TDF versus ETV for the treatment of HBV-ACLF. The primary outcome showed that TDF was comparable to ETV in terms of the survival rates of patients with HBV-ACLF, and the secondary results demonstrated that TDF was as effective as ETV in reducing HBV DNA and hepatic biochemical responses and may be more beneficial in improving liver function in the early stage of antiviral therapy.

To date, studies on the efficacy of TDF in HBV-ACLF are limited. Wan et al. [21] showed that TDF was superior to ETV in the treatment of HBV-ACLF in terms of rapid viral suppression within 2 weeks, improvement in liver function, and 48-week survival. In contrast, Li et al. [20] reported that compared to ETV, TDF in HBV-ACLF had a treatment response and clinical outcomes similar to those of ETV. Furthermore, at week 4, there was no significant difference in renal safety between these two treatment groups. The results of our meta-analysis are consistent with those reported by Li et al. [20] for TDF and ETV in terms of short-term virologic suppression and biomarkers of liver and kidney function. Although there was no significant difference in transplantation-free survival at 48 weeks, long-term follow-up is needed to determine the virologic response to TDF in these patients. Similar results were reported in other studies focused on the efficacy of TDF and ETV in chronic hepatitis B (CHB). Some meta-analyses showed that TDF had a greater ability to inhibit HBV and ETV can better normalize the ALT levels in the early stage, but there was no significant difference in long-term therapy. Additionally, TDF and ETV presented similar HBeAg clearance and seroconversion [27–29].

Since renal dysfunction is the most frequent complication in ACLF, the nephrotoxicity of therapeutic drugs is an important reference for clinical drug selection [3]. The nephrotoxicity of TDF initially raised concerns because of its structural similarity to adefovir, which is known to be nephrotoxic [30]. Both TDF and ETV are NAs that induce nephrotoxicity by mechanisms including renal tubular damage and mitochondrial toxicity [31]. Notably, in our study, TDF showed an unfavorable renal safety trend even in short-term treatment, although there was no significant difference between the two groups in terms of 4-week renal function changes. A recent real-world study in Korea indicated that TDF treatment reduced overall renal function in patients with CHB during the first 2 years [32]. Another systematic review based on 21 studies indicated that patients treated with TDF were not more likely to show renal function alteration than those treated with ETV. However, the eGFRs of patients receiving TDF tended to be more significantly decreased than those of patients receiving ETV [33]. Summarily, TDF and ETV are not contraindications in patients with underlying renal disease, but patients should be monitored closely due to the high risk of associated adverse effects. The dose of drugs should be adjusted according to the eGFR [34]. In our meta-analysis, TDF was reported to be more nephrotoxic than ETV. However, the significance of toxicity differences requires further investigation. Therefore, long-term follow-up may be useful to understand renal impairment in patients with ACLF receiving different antiviral therapies.

The limitations of this meta-analysis are as follows. First, only five studies were eligible, and four of them were prospective cohort studies without relevant randomized controlled trials (RCTs). All five studies were based on the Asian population, which may cause bias. Considering the high prevalence of HBV in other areas (e.g., sub-Saharan Africa), data from these regions are essential. Second, in our analysis, only two studies compared 48-week survival rates in both groups, and only one study consecutively reported changes in patients’ renal function over 48 weeks, so there was insufficient evidence to comprehensively and systematically assess the efficacy and safety of TDF and ETV. In addition, some data were extracted from the graphs provided in the text and may not be precise enough, as some studies did not provide raw data. Finally, our analysis mainly covered a period of up to 48 weeks, and a longer comparison of the efficacy of the two approaches is needed.

## Conclusion

In summary, our results suggest that TDF treatment of HBV-ACLF is similar to ETV in improving survival, liver function, and virologic response ETV, while the difference in nephrotoxicity needs further investigation. In the future, more studies are necessary, especially RCTs.

### Supplementary Information

**Additional file 1.**

## Abbreviation

ACLF: Acute-on-chronic liver failure
TDF: Tenofovir
ETV: Entecavir
CTP: Child-Turcotte-Pugh
MELD: Model for end-stage liver disease
NAs: Nucleotide analogues
eGFR: estimated Glomerular filtration rate
ALT: Alanine aminotransferase
TBiL: Total bilirubin
RR: Risk ratio
CI: Confidence interval
SMD: Standardized mean difference
RCTs: Randomized controlled trials
HBV: Hepatitis B virus
CHB: Chronic hepatitis B
HBV-ACLF: Acute-on-chronic liver failure associated with hepatitis B virus
