# Supplementary material for: Efficacy and safety of tenofovir disoproxil fumarate versus entecavir in the treatment of acute-on-chronic liver failure with hepatitis B: a systematic review and meta-analysis
Source: BMC Gastroenterol. 2023 Nov 13;23:388. doi: 10.1186/s12876-023-03024-7 (PMC10642028; doi:10.1186/s12876-023-03024-7)
Supplement: Supplementary file 1 — Additional file 1. [file 12876_2023_3024_MOESM1_ESM.docx]

**Supplementary Table 1: Search strategy used in databases**

| **Pubmed** | **Search items** |
| --- | --- |
| #1 | Entecavir (Title/Abstract) OR ETV (Title/Abstract) |
| #2 | Tenofovir (Title/Abstract) OR tenofovir disoproxil fumarate (Title/Abstract) OR TDF (Title/Abstract) |
| #3 | #1 OR #2 |
| #4 | Acute-On-Chronic Liver Failure[MeSH] OR ACLF (Title/Abstract) |
| #5 | Hepatitis B [MeSH] OR Hepatitis B Virus [MeSH] OR HBV (Title/Abstract) |
| #6 | #3 AND #4 AND #5 |
| **Embase** |  |
| #1 | Entecavir*:ab,ti OR ETV*:ab,ti |
| #2 | Tenofovir*:ab.ti OR tenofovir disoproxil fumarate*:ab,ti OR TDF*:ab,ti |
| #3 | #1 OR #2 |
| #4 | ‘Acute-On-Chronic Liver Failure’/exp |
| #5 | ACLF*:ab,ti |
| #6 | #4 OR #5 |
| #7 | ‘Hepatitis B’/exp |
| #8 | Hepatitis B virus*:ab,ti OR HBV*:ab,ti |
| #9 | #3 AND #6 AND #9 |
| **Web of Science** |  |
| #1 | TS=(Tenofovir) OR TS=(tenofovir disoproxil fumarate) OR TS=(TDF) |
| #2 | TS=(Entecavir) OR TS=(ETV) |
| #3 | #1 OR #2 |
| #4 | TS=(hepatitis B virus) OR TS=(hepatitis B) OR TS= (HBV) |
| #5 | TS=(Acute-On-Chronic Liver Failure) OR TS= (ACLF) |
| #6 | #3 AND #4 AND #5 |
| **Cochrane Library** |  |
| #1 | (tenofovir* OR tenofovir disoproxil fumarate * OR TDF*):ti,ab,kw |
| #2 | (entecavir* OR ETV*):ti,ab,kw |
| #3 | #1 OR #2 |
| #4 | MeSH descriptor: [Hepatitis B virus] explode all trees |
| #5 | MeSH descriptor: [Acute-On-Chronic Liver Failure] explode all trees |
| #6 | #3 AND #4 AND #5 |
